# Antifungal Drug Efficacy Profiles Against Vaginal *Candida albicans*: A Multi-Drug Comparative Analysis

**DOI:** 10.3390/jcm14207266

**Published:** 2025-10-15

**Authors:** Mohammad Zubair, Yazeed Albalawi

**Affiliations:** 1 Department of Medical Microbiology, Faculty of Medicine, University of Tabuk, Tabuk 47713, Saudi Arabia; 2 Molecular Microbiology and Infectious Disease Research Unit, University of Tabuk, Tabuk 47713, Saudi Arabia; 3 Department of Obstetrics and Gynecology, Faculty of Medicine, University of Tabuk, Tabuk 47713, Saudi Arabia

**Keywords:** vulvovaginal candida, *Candida albicans*, antifungal resistance, MIC_50_, MIC_90_, MFC, antifungal susceptibility testing, Saudi Arabia

## Abstract

**Background:** *Candida albicans* infects most reproductive-aged women, causing a prevalent infection known as Vulvovaginal *Candida*. As there has been an increase in resistance to widely used antifungal agents, particularly fluconazole, used in infections, local susceptibility profiles are needed to inform treatment options. **Methods:** This comparative observational study was carried out to determine the in vitro susceptibility of six antifungal compounds [fluconazole, voriconazole, itraconazole, ketoconazole, nystatin, and amphotericin B] to 163 vaginal *Candida albicans* isolates obtained in three hospitals in Tabuk, Saudi Arabia. MIC_50_, MIC_90_, and MFC values were calculated in Broth microdilution tests according to the standards of CLSI M27-A3. Friedman and Wilcoxon signed-rank tests were used to carry out statistical analysis. **Results:** It was observed that Amphotericin B and itraconazole recorded the lowest MIC and MFC, revealing better antifungal action. The worst performer was fluconazole with MIC_50_ (13.79 μg/mL), MIC_90_ (27.59 μg/mL), and MFC (37.93 μg/mL), and 85% resistance. It was found that there are significant differences between antifungal agents (*p* < 0.001), and amphotericin B and itraconazole always performed best compared to fluconazole and voriconazole. **Conclusions:** The results shows antifungal effectiveness as Amphotericin B and itraconazole are the most effective against vaginal *Candida albicans* isolates. There is a high rate of resistance to fluconazole, suggesting it should no longer be the first choice of treatment in this area. These findings highlight the need for local monitoring of drug resistance to guide treatment choices and emphasize the importance of using antifungals properly to prevent increased resistance.

## 1. Introduction

Vulvovaginal Candidiasis (VVC) is one of the most widespread genital infections in reproductive-age women in Western and high-income countries, with an estimated 70–75% of women having at least one episode in their lifetime, and 5–10% developing recurrent VVC (RVVC), that is, almost four or more episodes per year [1,2,3]. *Candida albicans* is still the most frequently occurring etiologic agent, causing 85–95% of symptomatic cases. Still, there is an increasing tendency in non-albicans Candida species, which tend to exhibit a higher level of antifungal resistance and are subject to treatment failure [4,5]. Vulvovaginal candidosis is a disease that affects women irrespective of immunocompetence and is associated with a considerable level of morbidity, which impacts the quality of life and healthcare resources on a worldwide level [6].

However, although the older generation of azoles (fluconazole) has been used as a first-line agent of VVC treatment over the years, emergent resistance is continuing to be reported. Thus, worldwide evidence shows that rates of fluconazole resistance among isolates of *Candida albicans* may vary, between ~2% in the UK and Ethiopia, and beyond 20% in Turkey, with a further increased value when vaginal pH becomes acidic [6,7]. The resistance levels were heterogeneous when isolates were analyzed in Thailand, Iran, and China [6,8,9], highlighting the importance of the different geographies’ surveillance [6,8,9]. In Peshawar, Pakistan, more than two in three (62%) Candida isolates were found to be fluconazole-resistant, but voriconazole had 85% susceptibility [10]. On the contrary, the research on Lebanon, Iran, and Vietnam showed reduced resistance (<6.3%) but confirmed the efficacy of amphotericin B and echinocandins [11]. This has brought out the geographic variations and the requirement of region-specific susceptibilities. The management problem and pathogenic spectrum of VVC are exacerbated in the case of the prevalence of non-albicans Candida species. In others, non-albicans *Candida* (e.g., *C. glabrata, C. krusei*) have come to make up as much as 45% of isolates and are inherently less susceptible to azoles [12,13]). Another study conducted in Indian multicentre centres demonstrated that voriconazole had better in vitro performance (91.6% sensitivity in *Candida albicans*) compared to fluconazole (also 91.6%), whereas amphotericin B and nystatin did not lose their activities in various species [12]. With these changes in resistance patterns, it is essential to have strong comparative analyses to demonstrate the effectiveness of antifungals. Recent studies have assessed susceptibility through MIC_50_, MIC_90_, and MFC assays of different agents [14,15]. Although various experiments have used the same complementary conditions, showing amphotericin B and itraconazole as more biologically active than fluconazole and ketoconazole in vitro [16], Moreover, the majority of published research is conducted in Europe, Asia, or Africa [10,12]. Still, very little information is available on Saudi Arabia. At the genetic level, there is a limited number of studies evaluating antifungal drug resistance among Candida isolates in Saudi Arabia; the findings concerning *Candida albicans* vaginal isolates, including MIC_50_, MIC_90_, and MFC, have not been published. These gaps obstruct evidence-based selection of antifungal agents in this region. This study aims to bridge the gap by conducting a comparative analysis of six antifungal agents: fluconazole, voriconazole, itraconazole, ketoconazole, nystatin, and amphotericin B against 163 clinical vaginal Candida albicans isolates collected in public hospitals in Tabuk City, Kingdom of Saudi Arabia. We will use broth microdilution according to CLSI M27-A3 to establish the value of MIC_50_, MIC_90_, and MFC of each agent.

This study aims to determine and compare the MIC_50_, MIC_90_, and MFC of six antifungal agents against vaginal *Candida albicans* isolates. This addresses a gap in the critical need for evidence-based and regionally applicable antifungal susceptibility data. With increasing antifungal resistance patterns and limited development of new agents, understanding the comparative efficacy of currently available drugs is essential for making effective treatment decisions in Saudi Arabia and other regions with similar epidemiological patterns.

## 2. Materials and Methods

### 2.1. Study Population

The sample consisted of reproductive women with clinical signs suggestive of vulvovaginal candida (VVC). These patients were referred for laboratory testing to three major healthcare facilities in Tabuk City, Saudi Arabia: the Maternity and Children’s Hospital (MCH-Tabuk), the University Medical Clinic, and Prince Fahad Bin Sultan Hospital. Patient enrolment followed over six months, from February 2025 to July 2025. Following routine vaginal examinations and specimen collection, swabs were cultured, and only those showing identification of *Candida albicans* were included in the study. A total of 163 confirmed *Candida albicans*-positive isolates were collected and analyzed. All participants were tested following standardized clinical procedures, and written informed consent was obtained according to the ethical standards.

### 2.2. Study Design and Setting

A comparative observational study of antifungal investigations on six commonly used antifungal agents was conducted in a laboratory to investigate and compare the antifungal efficacy profiles of these agents against clinically isolated *Candida albicans* vaginal isolates. Patients at three government health institutions in Tabuk City, Saudi Arabia, were surveyed at the Maternity and Children’s Hospital (MCH-Tabuk), the University Medical Clinic, and Prince Fahad Bin Sultan Hospital. The survey was conducted over six months, from February 2025 to July 2025. This study was approved by the Local Research Ethics Committee (UT-517-334-2025), under the aegis of the National Research Ethics Committee of the Kingdom of Saudi Arabia. All participants provided written informed consent before specimen collection, and patient confidentiality was maintained throughout the study, in accordance with the Declaration of Helsinki and national research ethical requirements.

### 2.3. Sample Size Estimation

The sample size was calculated using G* Power software version 3.1.9.4 [17]. When the one-way ANOVA (Fixed effects, omnibus) is considered with six groups and assuming a big effect size (f = 0.40), type I error rate (alpha = 0.05), and a statistical power of 0.95, the sample size needed was estimated to be 132 *Candida albicans* isolates (about 22 per group). The current analysis contains a total of 163 vaginal *Candida albicans*-positive isolates, and it surpasses the minimum requirement of 150 isolates.

### 2.4. Inclusion and Exclusion Criteria

Female participants of reproductive age were included in this study. Initially, the patients had to be referred for inclusion by having one or more characteristic signs of VVC (e.g., abnormal vaginal discharge, itching vulva, painful micturition, or swelling of the vulva) [18]. However, culture-confirmed *Candida albicans* isolates were considered only as final ones, which ensured that all cases met the clinical and microbiological requirements. Before participation, all subjects provided written informed consent.

The exclusion criteria were strictly followed to ensure that the study population was particular and any confounding factors were minimized. The patients were excluded in cases of non-albicans Candida, mixed fungal and bacterial vaginal infections, or pregnancy. Other exclusions were patients whose history included repeat VVC (four or more incidences of VVC in the last year), immunocompromised patients (e.g., HIV-positive, receiving chemotherapy or systemic immunosuppressive medication), or patients who received systemic or topical antifungal treatment within two weeks of sample collection. These criteria guaranteed a homogeneous group to assess the comparative effectiveness of antifungal drugs against *Candida albicans*, particularly.

### 2.5. Sample Collection and Processing

The patients at three local public healthcare facilities in Tabuk, Saudi Arabia (Maternity and Children Hospital (MCH- Tabuk), University Medical Clinic, and Prince Fahad Bin Sultan Hospital) with signs and symptoms of VVC were included in the study during the study period, February to July 2025, and had vaginal swab samples taken. Trained clinicians carried out Vaginal swabs, which were obtained aseptically, in case of symptoms of VVC in patients. The swabs were carried out to Sabouraud Dextrose Broth (SDB, Oxoid Ltd., Basingstoke, UK) within 2 h of collection and inoculated onto Sabouraud Dextrose Agar (SDA) to which chloramphenicol was added. Plates were incubated at 37 °C for 24–48 h. Typical Candida morphology colonies were identified to species-level by:

- Germ tube test (using horse serum, Sigma-Aldrich, St. Louis, MO, USA) [19]
- Chromogenic agar (CHROMagar Candida, CHROMagar, Saint-Denis, France) [20]
- Carbohydrate assimilation test (API 20C AUX, bioMérieux, Craponne, France) [20]

In order to obtain reliability of identification and antifungal susceptibility outcomes, reference quality control (QC) strains were added, *Candida albicans* ATCC 90028, *Candida parapsilosis* ATCC 22019, and *Candida krusei* ATCC 6258, as per CLSI recommendations [20,21,22,23]. Antifungal susceptibility test was performed according to CLSI M27-A3 broth microdilution. The antifungal reagents tested were: Fluconazole, Voriconazole, Itraconazole, Ketoconazole, Amphotericin B (all from Sigma-Aldrich, USA), and Nystatin (HiMedia, Mumbai, India). Minimal inhibitory concentration (MIC_50_, MIC_90_) and minimal fungicidal concentration (MFC) were determined for each antifungal agent. MIC breakpoints and interpretive criteria were applied using CLSI guidelines. MFCs were considered the lowest concentration, resulting in a ≥99.9% reduction in CFU compared to the initial inoculum.

### 2.6. Antifungal Susceptibility Testing

Antifungal susceptibility profiles were observed using the broth microdilution method following Clinical and Laboratory Standards Institute (CLSI) M27-A3 guidelines. The antifungal agents that were tested include Fluconazole, Voriconazole, Itraconazole, Ketoconazole, Nystatin, and Amphotericin B. Each isolate was analyzed for Minimum Inhibitory Concentration at 50% (MIC_50_), Minimum Inhibitory Concentration at 90% (MIC_90_), and Minimum Fungicidal Concentration (MFC). MICs were interpreted according to the CLSI cutoff point, where obtainable. MFCs were determined by subculturing 100 µL from wells with no visible growth onto fresh SDA plates and assessing colony formation after 48 h. The lowest concentration resulting in ≥99.9% reduction in colony-forming units was defined as the MFC.

### 2.7. Statistical Analysis

The statistical analysis procedures were conducted on IBM SPSS Statistics version 27.0. Descriptive statistics were used for the demographic characteristics. The Friedman test was applied to compare MIC_50_, MIC_90_, and MFC values among the six antifungal agents in a general comparison. In cases where there was a significant difference, pairwise comparisons were made using the Wilcoxon signed-rank test to determine the differences among specific groups. The Bonferroni correction was applied to adjust for the problem of multiple comparisons using a *p*-value of <0.05 as a significance level. The Bonferroni correction calculates adjusted significance levels using the formula 0.05/*n*, where *n* is the number of comparisons made. With 15 comparisons, the adjusted significance level was 0.05/15 = 0.0033. Thus, the statistical significance was considered only when the *p*-values were less than 0.0033 in pair comparison.

## 3. Results

There were 163 patients included in the study, with a Mean age of 37.10 ± 5.81 years, and the Body Mass Index (BMI) of the participants in the study was 20.31 ± 5.22. About the characteristics of vaginal discharge, most of the participants reported creamy yellow discharge 78, 47.9%), white discharge in 55 (33.7%), and off-white discharge in 30 (18.4%). Regarding the quantity of discharge, 96 (58.9%) of them discharged less, 60 (36.8%) had heavy discharge, and only 7 (4.3%) had medium discharge. The presence of itching in the vulva region was found in 78 (47.9%) of the participants, while 85 (52.1%) did not report any itching. Most of the participants (59.5%) complained of burning micturition. Similarly, 60.7% of the participants exhibited swelling of the vulva. The average duration of infection was 27.72 ± 22.05 days (Table 1).

**Figure 1:**
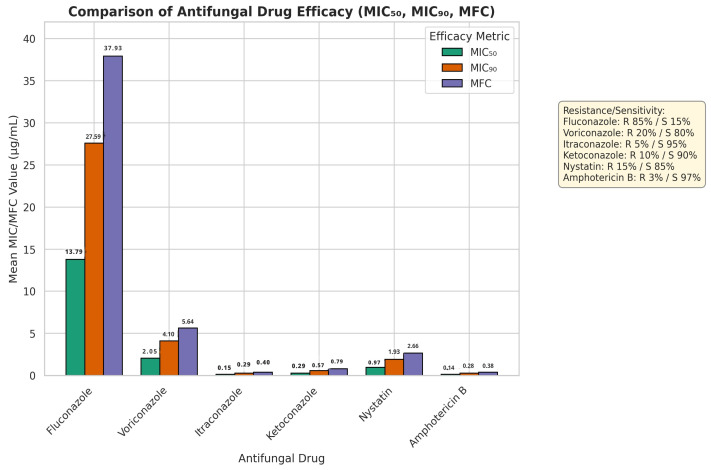
Comparative MIC_50_, MIC_90_, and MFC values of antifungal drugs against *Candida albicans* illustrating the relative potency of six antifungal agents (fluconazole, voriconazole, itraconazole, ketoconazole, nystatin, and amphotericin B).

Figure 1 displays the comparison of the relative potency of six antifungal agents (Fluconazole, Voriconazole, Itraconazole, Ketoconazole, Nystatin and Amphotericin B), in terms of MIC_50_, MIC_90_ and MFC. MIC_50_ (13.79 mg/mL), MIC_90_ (27.59 mg/mL) and MFC (37.93 mg/mL) of fluconazole were the highest of all the agents. Voriconazole reported the MIC_50_, MIC_90_, and MFC of 2.05 mg/mL, 4.10mg/mL, and 5.64mg/mL, respectively. The MIC_50_, MIC_90_ and MFC of Itraconazole were 0.15 mg/mL, 0.29 mg/mL and 0.40mg/mL, respectively. Ketoconazole had MIC_50_ of 0.29 mg/mL, MIC_90_ of 0.57 mg/mL and MFC of 0.79mg/mL. Amphotericin B displayed the lowest values in all the efficacy parameters (MIC_50_: 0.14 mg/mL, MIC_90_: 0.28mg/mL, MFC: 0.38 mg/mL). Nystatin had relatively low MIC and MFC values also.

**Figure 2:**
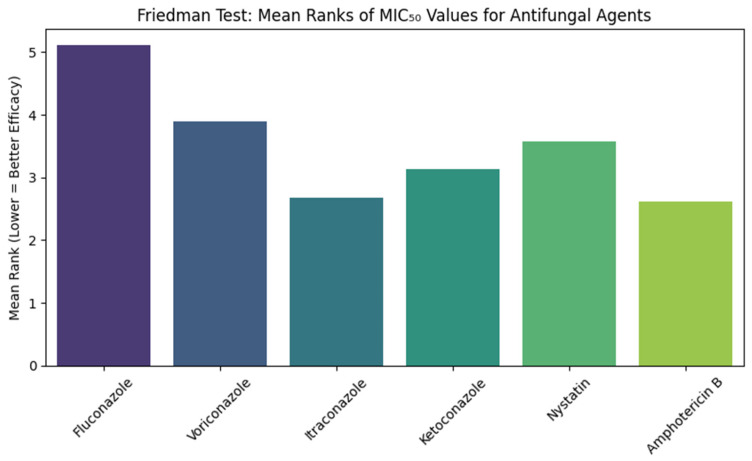
Friedman Test Comparison of MIC_50_ Values of Antifungal Agents Against *Candida albicans* showing the statistical comparison of MIC_50_ values using the Friedman test. Lower Mean ranks correspond to higher antifungal activity, with amphotericin B and itraconazole demonstrating superior efficacy compared to fluconazole.

Figure 2 shows the results of the Friedman test comparing the Minimum Inhibitory Concentration (MIC) of six antifungal agents against *Candida albicans* isolates. The test showed a significant difference between the antifungal agents, with chi-squared values of 217.354 (df = 5, *p* < 0.001). The Mean rank for fluconazole was the highest (5.11), while Amphotericin B and Itraconazole showed the lowest Mean ranks (2.62 and 2.67, respectively). Voriconazole, Ketoconazole, and Nystatin with Mean ranks of 3.9, 3.13, and 3.58, respectively.

**Figure 3:**
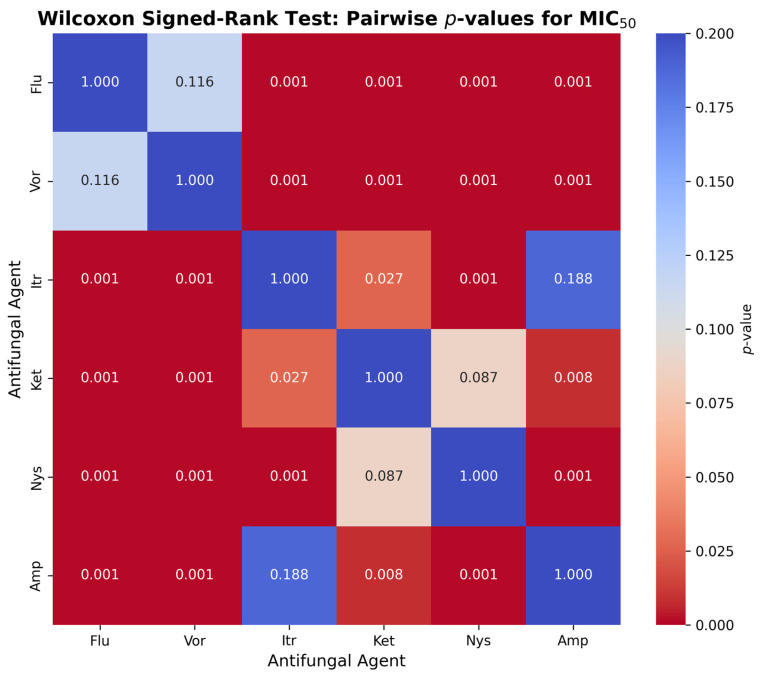
Pairwise Comparison of MIC_50_ Values of Antifungal Agents Using Wilcoxon Signed-Rank Test illustrating pairwise statistical comparisons between antifungal agents. Significant *p*-values (*p* < 0.05 after Bonferroni correction) indicate differences in antifungal activity, highlighting the superiority of Amp (amphotericin B) and Itr (itraconazole) over Flu (fluconazole) and Vor (voriconazole).

Figure 3 presents the pairwise comparison with the Wilcoxon signed-rank test to determine the difference in values of the MIC_50_ between the six antifungal agents and *Candida albicans*. Compared to Itraconazole (*p* = 0.0001), Ketoconazole (*p* = 0.0001), Nystatin (*p* = 0.0001) and Amphotericin B (*p* = 0.0001), Fluconazole significantly exceeded MIC_50_ values (*p* = 0.0001), but not significantly surpassed Voriconazole (*p* = 0.116). Voriconazole showed much higher MIC_50_ values than Itraconazole, Ketoconazole, Nystatin and Amphotericin B (*p* < 0.001). Ketoconazole had greater MIC_50_ values than Itraconazole (*p* = 0.027), Amphotericin B (*p* = 0.008), but not Nystatin (*p* = 0.087). The MIC_50_ of Nystatin was higher than Itraconazole (*p* < 0.001) and Amphotericin B (*p* < 0.001). Itraconazole and Amphotericin B were not found to be significantly different (*p* = 0.188).

**Figure 4:**
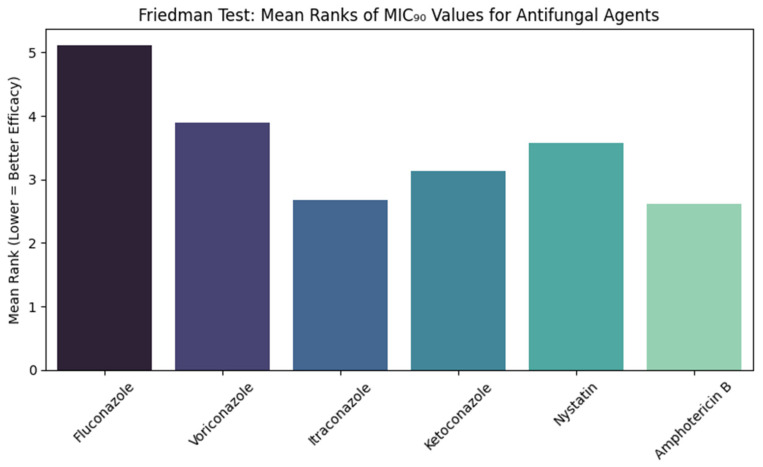
Friedman Test Comparison of MIC_90_ Values of Antifungal Agents Against *Candida albicans* showing the Friedman test results for MIC_90_ values, demonstrating significant variation in antifungal efficacy. Amphotericin B and itraconazole consistently had the lowest Mean ranks, indicating stronger inhibition of *Candida albicans* growth.

Figure 4 shows the comparisons of the MIC_90_ values of six antifungal agents against *Candida albicans* using the Friedman test. The test showed a statistically significant result (χ^2^ = 217.354, df = 5, *p* < 0.001). The Mean rank of fluconazole was the highest (5.11), while amphotericin B (2.62) and itraconazole (2.67) had the lowest Mean ranks. Voriconazole, ketoconazole, and nystatin Mean ranks were 3.9, 3.13, and 3.58, respectively.

**Figure 5:**
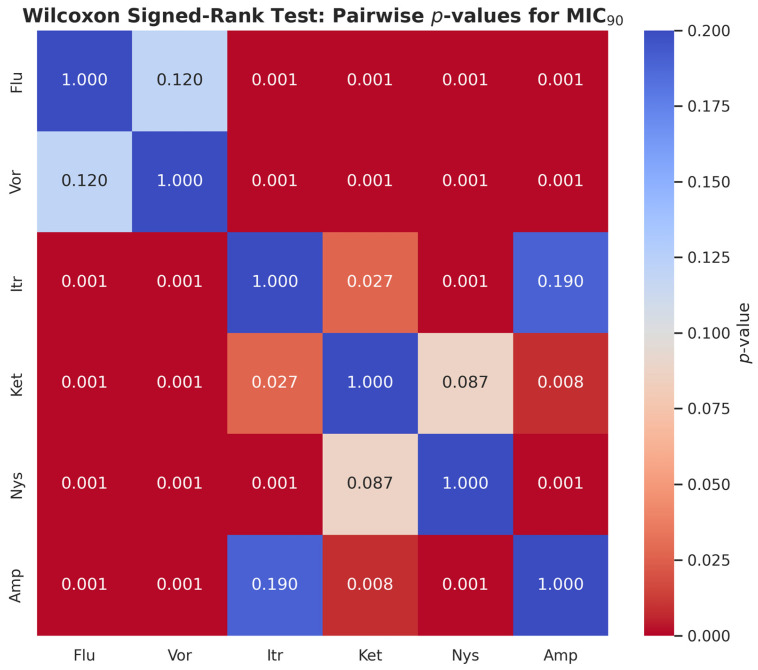
Pairwise Comparison of MIC_90_ Values of Antifungal Agents Using Wilcoxon Signed-Rank Test presenting pairwise statistical comparisons of MIC_90_ values. Amp (Amphotericin B) and Itr (itraconazole) demonstrated significantly lower MIC_90_ values than Flu (fluconazole) and Vor (voriconazole), confirming their higher antifungal potency.

Figure 5 presents the two-tailed comparisons between MIC_90_ values of antifungal agents against *Candida albicans*, which have been performed with the Wilcoxon signed-rank test. Compared to Fluconazole, itraconazole (*p* < 0.001), Ketoconazole (*p* < 0.001), Nystatin (*p* < 0.001), and amphotericin B (*p* < 0.001) had much lower MIC_90_ values. The Fluconazole and Voriconazole were not significantly different (*p* = 0.116). All MIC_90_ concentrations of Itraconazole, Ketoconazole, Nystatin and Amphotericin B were significantly lower as compared to voriconazole (*p* < 0.001). There was a considerable distinction between Itraconazole and Ketoconazole (*p* = 0.027). The MIC_90_ value was much higher in nystatin than in Itraconazole (*p* < 0.001). Amphotericin B had a much lower value of MIC_90_ than Ketoconazole (*p* = 0.008) and Nystatin (*p* < 0.001). There was no significant difference between Itraconazole and Amphotericin B (*p* = 0.188), or Nystatin and Ketoconazole (*p* = 0.087).

**Figure 6:**
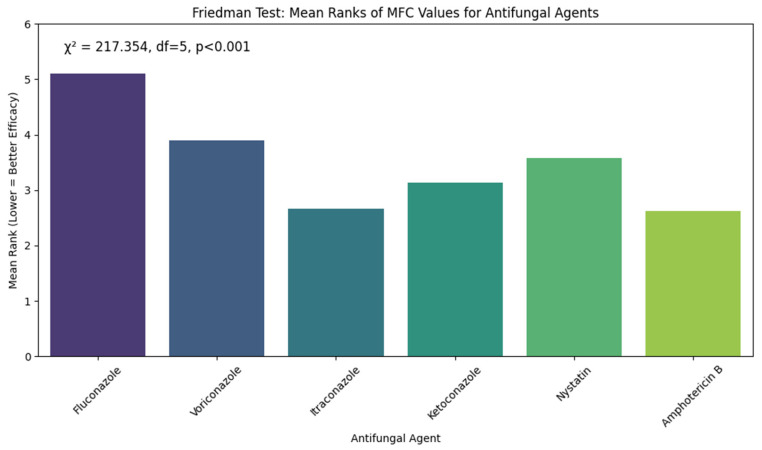
Friedman Test for Comparison of MFC Values of Antifungal Agents Against *Candida albicans* displaying the Friedman test results comparing fungicidal activity (MFC values). Amphotericin B and itraconazole showed the lowest Mean ranks, indicating superior fungicidal activity compared to fluconazole and voriconazole.

Figure 6 shows the results of the Friedman test to determine variations in the Minimum Fungicidal Concentration (MFC) values of 6 antifungal agents on *Candida albicans*. The test showed that the difference in the MFC values among the antifungal drugs was very significant, with the chi-square value = 217.354, degrees of freedom = 5, with *p*-value < 0.001. Fluconazole scored the highest Mean rank (5.11), showing that it also requires the best concentrations to illustrate fungicidal activity, reflecting the lower fungicidal activity and effectiveness. Amphotericin B (Mean rank = 2.62) and Itraconazole (Mean rank = 2.67) showed the lowest output, which implies great fungicidal activity at the low level. Other agents were between poor and good, with the MFC values of Voriconazole (3.9), Ketoconazole (3.13), and Nystatin (3.58). These results indicate that Amphotericin B and Itraconazole are the most fungicidal agents against *Candida albicans* in the current study.

**Figure 7:**
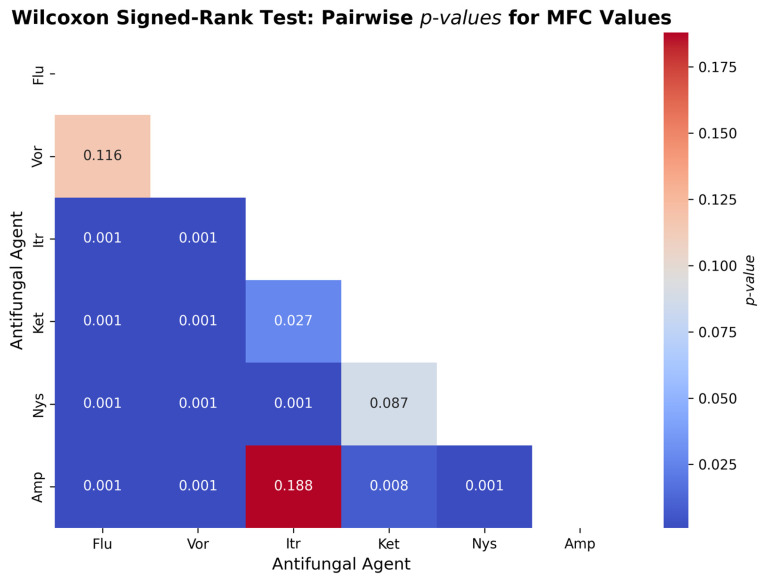
Pairwise Comparison of MFC Values of Antifungal Agents Using Wilcoxon Signed-Rank Test figure showing pairwise comparisons of fungicidal activity. Amp (Amphotericin B) and Itr (itraconazole) exhibited significantly lower MFC values than flu (fluconazole), vor (voriconazole), and ket (ketoconazole), highlighting their stronger fungicidal effect.

Figure 7 illustrates the outcome of the Wilcoxon signed-rank test that has been conducted to examine the difference in the Minimum Fungicidal Concentration (MFC) values of six antifungal agents examined on the *Candida albicans*. The comparison showed that there were some statistically significant differences between the antifungal drugs, meaning that they possess various levels of fungicidal activity. The MFC values of Itraconazole (*p* < 0.001), Ketoconazole (*p* < 0.001), Nystatin (*p* < 0.001), and Amphotericin B (*p* < 0.001) were also lower than that of Fluconazole, indicating that they are more fungicidal. There was no marked difference between Fluconazole and Voriconazole (*p* = 0.116), which means that fungicidal effect is similar. Subsequent comparisons revealed that Itraconazole was significantly less MFCs than Voriconazole, Ketoconazole and Nystatin (in *p* < 0.001, *p* = 0.027 and *p* < 0.001, respectively), indicating superior efficacy. Nonetheless, it was not statistically significant compared to Amphotericin B (*p* = 0.188), indicating that the fungicidal activity of these two agents is similar. Providing the key properties of Amphotericin B against other agents, it MFCs were cooked significantly lower than Voriconazole (*p* < 0.001), Ketoconazole (*p* = 0.008) and Nystatin (*p* < 0.001). There was no significant difference between Nystatin vs. Ketoconazole (*p* = 0.087) implying that the two have similar MFC values.

The results of the PERMANOVA test are presented in Table 2. The differences in overall patterns of antifungal sensitivity at MIC_50_, MIC_90_ and MFC were significantly related to fluconazole resistance (pseudo-F = 126.69, *p* = 0.001). Ketoconazole resistance (pseudo-F = 8.98, *p* = 0.004) and nystatin resistance (pseudo-F = 11.28, *p* = 0.005) also had significant effects. There were no significant differences in resistance of voriconazole and amphotericin B (*p* > 0.1 and *p* > 0.7, respectively). The itraconazole resistance was not possible to determine due to the lack of variation between the isolates (one category).

## 4. Discussion

The aim of the study was to evaluate the antifungal susceptibility among *Candida albicans* vaginal isolates in Tabuk, Saudi Arabia, against six widely used antifungal agents. The MIC_50_, MIC_90_, and MFC values showed a significant difference occurred in the drug potency, where Amphotericin B and Itraconazole had high antifungal activity, and Fluconazole was the least active and had the highest percentage of resistance. The identified high resistance to Fluconazole (85%) is in line with current world trends of decreased azole effectiveness. Resistance rates were high in Pakistan [10] and Vietnam [8], and Sobel [2] found an increasing resistance to fluconazole over the past decade among Candida isolates in vaginal specimens in the United States. This observation highlights that lower sensitivity to fluconazole is not only a problem associated with a geographically specific area [24]. Given that fluconazole remains the first-line treatment for VVC [3,24], the continually high resistance rates reported here and by other sources are a cause for legitimate concern regarding its use in clinical practice, especially in instances of recurrent infection.

Amphotericin B, and Itraconazole on the other hand displayed superior efficacy as observed in low MIC and MFC values and minimum resistance rates of 5%and 3%, respectively. The same findings were established by Rati et al. [12], Nguyen et al. [25], and Tan et al. [14], who reported that these agents have continued to show effectiveness against wild-type as well as azole-resistant *Candida albicans*. Amphotericin B possesses strong fungicidal effects, which are based on the specific mechanism of combination with the ergosterol and ruining the membrane integrity, allowing fungal cells to die quickly [15]. Itraconazole, which acts by a different mechanism, involving interference with ergosterol synthesis, is also very effective, especially in azole-resistant species. These results indicate that the two medications are still important treatment choices and can be used as alternative agents where UF-resistant VVC is present.

Itraconazole, Ketoconazole, and Nystatin showed intermediate activity in our study. Voriconazole had a moderate effect, which aligned with the study by Rati et al. [12], who noted that although it was more effective than fluconazole, it was not as potent as Itraconazole and Amphotericin B. Ketoconazole and Nystatin demonstrated relatively low MIC and MFC levels. These findings resonated with reports made by Hui et al. [26], Mirshekar et al. [27], and Araújo et al. [28]. However, these agents have limited clinical application as systemic Ketoconazole has been discouraged because of its toxicity, and Nystatin does not penetrate as well systemically [5].

Notably, the fact that the findings are based on statistical analyses enhances their validity. The Friedman test confirmed significant between-group differences across the antifungal agents (χ^2^ = 217.354, *p* < 0.001), and Wilcoxon signed-rank tests were always substantial to indicate that Amphotericin B and Itraconazole were superior to fluconazole and voriconazole (*p* < 0.001 in all cases). The advanced non-parametric tests, PERMANOVA, also indicated that fluconazole, ketoconazole, and nystatin resistance were significantly correlated with overall susceptibility patterns, whereas resistance to voriconazole and amphotericin B was not. Despite the findings that amphotericin B and itraconazole showed greater in vitro effectiveness than other antifungal agents, particularly fluconazole, such translation cannot be performed with care for the clinical. Amphotericin B is a powerful agent, but with established nephrotoxicity and infusion-related adverse drug effects that restrict its application in the treatment of routine cases of vulvovaginal candidiasis [29,30]. Conversely, itraconazole is safer as an oral drug, though it can result in hepatotoxicity and drug–drug interactions and close observation when used in the long term or systemically [31]. Fluconazole remains a common treatment for VVC worldwide due to its low cost, oral route of administration, and favourable safety profile. The high rate of resistance observed, though, in this research indicates that the application of empirical fluconazole treatment in Tabuk is likely to result in treatment failure and recurrence of VVC. Therefore, although a change towards itraconazole or amphotericin B is successful, it should be weighed against the possible toxicity of it, cost, and its availability in the local healthcare system. These results illustrate the necessity of antifungal stewardship initiatives and cost-efficiency studies as a Means of informed sustainable therapy selection in regions with the growing prevalence of azole resistance. The use of this multidimensional statistical tool enhances the strength of our conclusions as well as emphasises the clinical significance of our in vitro findings.

The combined effect of these leads to the conclusion that there will be a change in the treatment landscape of VVC in Saudi Arabia. Although fluconazole has been historically the most prescribed antifungal, the high resistance rates in the present study indicate that it might not be suited anymore as the first-line choice in this region. Amphotericin B, Itraconazole, and, probably, Voriconazole, Ketoconazole, and Nystatin find their application in specific cases or preparations for topical application.

These results have great public health and clinical significance. However, there are some limitations that need to be considered. This study was implemented within one geographical area (Tabuk, Saudi Arabia), and this could be a weakness in generalising the results to the population with other antifungal use patterns or epidemiological characteristics. However, this regional coverage offers a much-needed baseline information to Saudi Arabia, where local antifungal resistance trends are largely underreported, and is an important addition to global surveillance efforts, which demonstrate geographic variability. The decision-making process was based on in vitro susceptibility testing, which was certainly informative, but may not be complete in clinical results where host, drug pharmacokinetics, and immune response are significant. Following an equally modest sample with a limitation to vaginal isolates of *Candida albicans* and not including non-albicans species of Candida, increasingly supported in the context of VVC and tending towards higher resistance. In addition, molecular characterization of resistance mechanisms was not conducted, which would have yielded further knowledge on the genetic and biochemical basis of antifungal resistance, and the cross-sectional nature did not allow checking temporal changes in resistance patterns. Future efforts should address these gaps with multicenter, longitudinal studies incorporating molecular profiling, providing a more detailed understanding of the dynamics of antifungal resistance.

## 5. Conclusions

In conclusion, this study showed that Amphotericin B and Itraconazole are the most efficient antifungal drugs against *Candida albicans* vaginal isolates in Tabuk, Saudi Arabia, due to their low MIC and MFC results. In contrast, Fluconazole had the highest resistance rates, and Voriconazole has partial effectiveness too. This evidence provides a compelling rationale for re-evaluating clinical practice with the use of azoles, particularly Fluconazole, in the treatment of VVC in this region.

In addition to local medical implications, the findings emphasize the need to retain continuous antifungal surveillance schemes that monitor changes in resistance patterns and inform subsequent sensible prescribing decisions. Moreover, the use of advanced statistical models, including PERMANOVA, enabled the identification of resistance-related differences in profiles of overall susceptibilities, and these data can be incorporated into projections of resistance evolution.

Future studies should comprise longitudinal, multicenter studies to confirm the findings, identify molecular mechanisms of resistance, and formulate personalized treatment guidelines that incorporate risk factors in the host and the resistance phenotype. Moreover, the findings can guide antifungal surveillance efforts and enable the design of newer antifungal agents or combination therapeutics that can combat this resistance.

## Figures and Tables

**Table 1:** Demographic Characteristics.

		Mean ± Standard Deviation	*n* (%)
Age		37.10 ± 5.81	-
BMI		20.31 ± 5.22	-
Discharge colour	Creamy yellow	-	78 (47.9%)
	Off White	-	30 (18.4%)
	White	-	55 (33.7%)
Discharge amount	Heavy		60 (36.8%)
	Less	-	96 (58.9%)
	Medium	-	7 (4.3%)
Vulval itching	No	-	85 (52.1%)
	Yes	-	78 (47.9%)
Burning micturition	No	-	66 (40.5%)
	Yes	-	97 (59.5%)
Vulval swilling	No	-	64 (39.3%)
	Yes	-	99 (60.7%)
Infection Duration (Days)		27.72 ± 22.05	

This table summarizes the baseline demographic and clinical characteristics of 163 women with vaginal *Candida albicans* infection, including age, BMI, type and amount of vaginal discharge, presence of vulval itching, burning micturition, vulval swelling, and average duration of infection. Data are presented as Mean ± SD or *n* (%).

**Table 2:** PERMANOVA results for antifungal resistance factors on MIC_50_, MIC_90_, and MFC values.

Resistance Factor	MIC_50_		MIC_90_		MFC	
	Pseudo-F	*p*-Value	Pseudo-F	*p*-Value	Pseudo-F	*p*-Value
Fluconazole	126.69	0.001	126.69	0.001	126.69	0.001
Voriconazole	1.65	0.138	1.65	0.117	1.65	0.149
Itraconazole	-	-	-	-	-	-
Ketoconazole	8.98	0.003	8.98	0.004	8.98	0.002
Nystatin	11.28	0.003	11.28	0.005	11.28	0.005
Amphotericin B	0.23	0.825	0.23	0.808	0.23	0.789

This table presents the effect of resistance to individual antifungal drugs on overall susceptibility patterns. Pseudo-F and *p*-values indicate whether resistance significantly influenced variation in MIC_50_, MIC_90_, and MFC values among isolates. Significant associations are shown for fluconazole, ketoconazole, and nystatin, while amphotericin B and voriconazole did not show significant effects.

## Data Availability

The datasets used and analyzed during the current study are available from the corresponding author on reasonable request.

## Abbreviations

The following abbreviations are used in this manuscript:

VVC	Vulvovaginal Candidiasis
RVVC	Recurrent Vulvovaginal Candidiasis
MIC_50_	Minimum Inhibitory Concentration required to inhibit 50% of isolates
MIC_90_	Minimum Inhibitory Concentration required to inhibit 90% of isolates
MFC	Minimum Fungicidal Concentration
CLSI	Clinical and Laboratory Standards Institute
SDA	Sabouraud Dextrose Agar
API 20C AUX	Analytical Profile Index 20C AUX (yeast identification system, bioMérieux)
SPSS	Statistical Package for the Social Sciences
ANOVA	Analysis of Variance
df	Degrees of Freedom
χ^2^	Chi-square
PERMANOVA	Permutational Multivariate Analysis of Variance
